# Transient Elastography and Ultrasonography: Optimal Evaluation of Liver Fibrosis and Cirrhosis in Patients with Chronic Hepatitis B Concurrent with Nonalcoholic Fatty Liver Disease

**DOI:** 10.1155/2019/3951574

**Published:** 2019-01-23

**Authors:** Geng-lin Zhang, Qi-yi Zhao, Chao-shuang Lin, Zhao-xia Hu, Ting Zhang, Zhi-liang Gao

**Affiliations:** ^1^Department of Infectious Diseases, The Third Affiliated Hospital of Sun-Yat-sen University, Guangzhou, China; ^2^Guangdong Provincial Key Laboratory of Liver Disease, The Third Affiliated Hospital of Sun-Yat-sen University, Guangzhou, China; ^3^Department of Ultrasound, The Third Affiliated Hospital of Sun-Yat-sen University, Guangzhou, China; ^4^Key Laboratory of Tropical Disease Control, Sun-Yat-sen University, Ministry of Education, Guangzhou, China

## Abstract

**Background and Aims:**

Concordance between transient elastography (TE) and ultrasonography (US) in assessing liver fibrosis in patients with chronic hepatitis B (CHB) and concurrent nonalcoholic fatty liver disease (NAFLD) has been rarely studied. This study aimed to evaluate the individual and combined performances of TE and US in assessing liver fibrosis and cirrhosis.

**Patients and Methods:**

Consecutive CHB patients with NAFLD were prospectively enrolled. TE and US examinations were performed, with liver biopsy as a reference standard. Receiver operating characteristic (ROC) curves were obtained to evaluate the diagnostic performance. Differences between the areas under the ROC curves (AUCs) were compared using DeLong's test.

**Results:**

TE and US scores correlated significantly with the histological fibrosis staging scores. TE was significantly superior to US in the diagnosis of significant fibrosis (AUC, 0.84 vs 0.73; P=0.02), advanced fibrosis (AUC, 0.95 vs 0.76; P<0.001), and cirrhosis (AUC, 0.96 vs 0.71; P<0.001). Combining TE with US did not increase the accuracy of detecting significant fibrosis, advanced cirrhosis, or cirrhosis (P=0.62, P=0.69, and P=0.38, respectively) compared to TE alone. However, TE combined with US significantly increased the positive predictive value for significant fibrosis when compared to TE alone. The optimal cut-off values of TE for predicting advanced fibrosis and cirrhosis were 8.7 kPa and 10.9 kPa, with negative predictive values of 92.4% and 98.7%, respectively.

**Conclusions:**

TE is useful for predicting hepatic fibrosis and excluding cirrhosis in CHB patients with NAFLD. A combination of TE and US does not improve the accuracy in assessing liver fibrosis or cirrhosis.

## 1. Introduction

Chronic hepatitis B (CHB) and nonalcoholic fatty liver disease (NAFLD) are chronic liver diseases with a high incidence worldwide [1, 2]. NAFLD has a spectrum comprised of fatty liver, nonalcoholic steatohepatitis (NASH), advanced fibrosis, and cirrhosis. CHB and NAFLD commonly cause cirrhosis and hepatocellular carcinoma (HCC) [3, 4]. Currently, the increasing rate of NAFLD in CHB patients is alarming [5]. A study found that NASH (a type of NAFLD) was independently correlated with liver fibrosis in patients with CHB [6]. Moreover, another cohort study found that concurrent fatty liver can independently increase hepatitis B virus (HBV)-related HCC development 7.3-fold [7]. These reports suggest that timely and accurate diagnosis of liver fibrosis in CHB patients with NAFLD is urgent. Moreover, the assessment of liver fibrosis in patients with chronic liver diseases, especially those with coetiologies, is mandatory and recommended by international practice guidelines. Liver biopsy (LB) has been the gold standard for assessing liver fibrosis [8]. However, it is invasive and may result in several complications [9]. These disadvantages make it impractical to be performed regularly in clinical practice. Therefore, accurate and noninvasive tools that can clinically assess liver fibrosis in CHB patients with NAFLD are urgently needed.

Abdominal ultrasonography (US) is performed on CHB patients to assess structural changes and screen for HCC. Several US signs, such as an uneven or undulating liver surface, irregular echotexture of the liver parenchyma, spleen size, and changes in the diameters of vessels, have been found to be correlated with liver cirrhosis [10, 11]. Transient elastography (TE) is an ultrasound-based technology measuring liver stiffness by the difference in velocity of elastic shear wave propagation across the liver. TE has been repeatedly validated and has shown overall good accuracy in evaluating fibrosis and cirrhosis in different settings [12]. However, TE could be influenced by patient-dependent factors, including liver inflammation, liver congestion, and biliary obstruction [12, 13]. Therefore, the results should be interpreted with accurate clinical information.

The existence of NAFLD may cause morphological changes in the liver of CHB patients, which may make it more difficult to accurately evaluate the degree of fibrosis. To our knowledge, no comparison between US and TE in assessing liver fibrosis in CHB patients with NAFLD has been previously reported. Thus, the aim of this study was to evaluate the individual and combined performances of TE and US in assessing liver fibrosis and cirrhosis and to determine when TE should be added to US in CHB patients with NAFLD, using histological evaluation as the reference standard.

## 2. Patients and Methods

### 2.1. Patients

Between July 2013 and February 2018, adult CHB patients with NAFLD who were consecutively admitted to our hospital to undergo LB were prospectively enrolled. CHB patients were diagnosed as those who displayed hepatitis B surface antigen (HBsAg) positivity for more than 6 months [14, 15]. NAFLD was defined by the presence of hepatic steatosis (≥5%) and the absence of a history of significant alcohol consumption (where absence is defined as alcohol intake <20 g/day for men and intake <10 g/day for women) and the absence of other etiologies that may cause hepatic steatosis [16–18]. The exclusion criteria included the following: age <18, body mass index (BMI) ≥30 kg/m^2^, aspartate aminotransferase (AST) or alanine aminotransferase (ALT) ≥5 times of the upper limit of normal (ULN), coinfection with another hepatitis virus or human immunodeficiency virus (HIV), concurrent tumors, receiving antiviral therapy, significant alcohol consumption, currently taking medications that may induce hepatic steatosis (including corticosteroids, methotrexate, and tamoxifen), and pregnancy. Blood samples were obtained on the day of LB examination. The following data were collected from all patients: age; sex; weight; height; ALT, AST, total bilirubin, albumin, alkaline phosphatase (ALP), gamma-glutamyl transpeptidase (GGT), fasting glucose, and lipid levels; platelet counts; and prothrombin time activity. BMI was calculated as the body weight (kg)/height^2^ (m^2^). The study was conducted in accordance with the Declaration of Helsinki, and the protocol was approved by our hospital's ethics committee. Informed consent was obtained from each patient.

### 2.2. Liver Histological Analysis

A US-guided percutaneous LB (length >15 mm) of the right lobe was performed using a 16-gauge Magnum needle (Bard, Tempe, AZ, USA). Specimens were fixed in formalin and embedded in paraffin. Liver histology was assessed separately by two liver pathologists with more than 10 and 20 years of experience without knowledge of the clinical data or the TE and US results. Liver fibrosis was staged on a 0 to 4 scale according to the METAVIR scoring system [19]: F0, no fibrosis; F1, portal fibrosis without septa; F2, portal fibrosis and few septa; F3, numerous septa without cirrhosis; F4, cirrhosis. Steatosis was defined as the percentage of fat in hepatocytes and was graded as follows: 0, steatosis <5%; 1, 5-33% steatosis; 2, 34-66% steatosis; 3, steatosis >66% [17].

### 2.3. Liver Stiffness Measurement (LSM) by TE

TE was performed with a FibroScan system (Echosens, Paris, France) using the M probe. After an overnight fast, patients underwent TE examination within 3 days of LB by trained operators who had previously performed at least 500 scans in patients with chronic liver disease. Operators were blinded to the clinical data and pathology results. The value expressed in kilopascal (kPa) was recorded as a representation of the LSM. Up to 10 valid measurements were performed on each patient. A success rate above 60% and an interquartile range/median ratio of less than 30% were considered reliable [20].

### 2.4. Abdominal Ultrasound Examination

**Figure 1:**
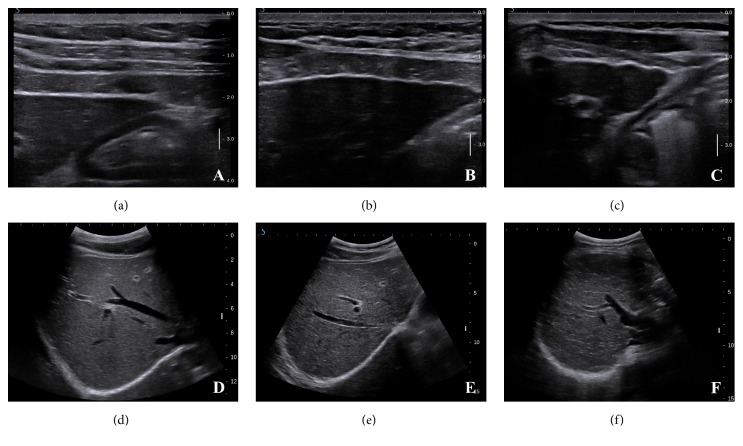
B-mode images of conventional ultrasonography (US) scoring system. (a) Smooth liver surface, score of 1. (b) Uneven liver surface, score of 2. (c) Irregular nodular liver surface, score of 3. (d) Homogeneous parenchyma, score of 1; and smooth hepatic vein vessel wall, score of 1. (e) Heterogeneous liver parenchyma with fine scattered hyperechoic or hypoechoic areas, score of 2. Obscured or slightly irregular hepatic vein vessel wall, score of 2. (f) Coarse liver parenchyma with an irregular pattern, score of 3.

All participants underwent a B-mode liver ultrasound scan within 1 week of LB. Each scan was performed using a Supersonic Imagine Aixplorer ultrasound system (Supersonic Imagine, Aix-en-Provence, France) equipped with an SC6-1 convex array probe with a frequency of 1-6 MHz. Two sonographers (with 10 years and 15 years of US experience) reviewed the B-mode images independently to assess the interobserver agreement without knowledge of the TE results or clinical data, but the final results were obtained in consensus to assess the diagnostic performance. A US scoring system developed in previous studies [10, 11] was used to evaluate the degree of liver cirrhosis (Figure 1). The scoring system included the following signs and scores: the liver surface (1 for a smooth surface, 2 for an uneven or wavy surface, and 3 for an irregular nodular surface); the liver parenchyma (1 for homogeneous parenchyma, 2 for heterogeneous parenchyma with fine scattered hyperechoic or hypoechoic areas, and 3 for coarse liver parenchyma with an irregular pattern); hepatic vein contour (1 for a smooth vessel wall, 2 for an obscured or slightly irregular vessel wall, and 3 for an irregular vessel wall with a narrowed diameter); a spleen index, calculated as the product of the oblique and diagonal diameters (1 for product <20cm^2^ and 2 for product >20cm^2^). The US scores ranged from 4 for a normal liver to 11 for advanced liver cirrhosis, and the scores were recorded in a standard form.

### 2.5. Virological Analyses

Serum HBV markers, including HBsAg, hepatitis B e antigen (HBeAg), and hepatitis B e antibody (HBeAb), were determined using the Elecsys system (Hoffmann-La Roche, Basel, Switzerland). Serum HBV-DNA levels were quantified by Cobas TaqMan (Hoffmann-La Roche, Basel, Switzerland) according to the manufacturer's instructions. The limit of detection of the assay was 20 IU/ml.

### 2.6. Statistical Analysis

Statistical analyses were performed using SPSS version 20.0 software (Chicago, IL, USA) and MedCalc version 15.2.2 (MedCalc Software, Mariakerke, Belgium). The data are expressed as frequencies, medians and ranges or means and standard deviations, as appropriate. Differences in TE were analyzed using Student's t-tests. The interobserver reproducibility of US scores was assessed by calculating the intraclass correlation coefficient. Spearman correlation coefficients were used to analyze the correlation between TE, US scores, hepatic steatosis, and fibrosis stage, and the correlation coefficients were compared using Fisher's* Z *test. Receiver operating characteristic (ROC) curves were used to assess the overall accuracy and to identify optimal cut-off values. The optimal cut-off values were selected according to Youden's index. The sensitivity, specificity, positive predictive value (PPV), negative predictive value (NPV), and positive likelihood ratio (PLR) and negative likelihood ratio (NLR) were calculated with 95% confidence intervals (CIs). Differences between the areas under the ROC curves (AUCs) were compared using DeLong's test. A 2-tailed P <0.05 was considered statistically significant.

## 3. Results

### 3.1. Characteristics of CHB Patients with NAFLD

**Figure 2:**
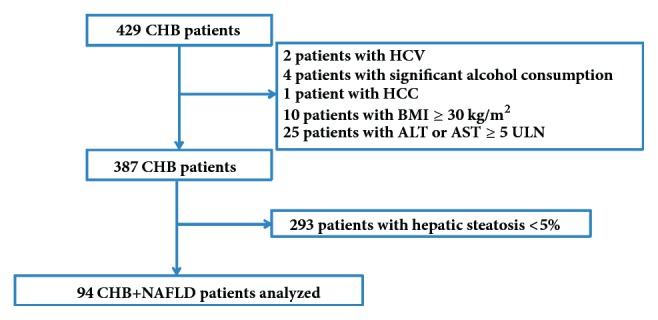
Selection and deposition of patients.

We included 429 consecutive, treatment-naive CHB patients who underwent TE, US, and LB. Forty-two patients were excluded for the following reasons: 2 patients were coinfected with hepatitis C virus (HCV), 4 patients had a history of significant alcohol consumption, 1 patient had concurrent HCC, 10 patients had a BMI ≥30 kg/m^2^, and 25 patients had ALT or AST levels ≥5 times of ULN. Among the remaining patients, 293 patients did not have NAFLD (hepatic steatosis <5%), and 94CHB patients had NAFLD (hepatic steatosis ≥5%), resulting in a rate of NAFLD in CHB patients of 24.3% (94/387). A summary of patients' deposition is shown in Figure 2. Among the CHB patients with NAFLD, they had a mean age of 36.90 years; only one patient had hypertension, and none had diabetes mellitus. 29 (30.9%) patients had at least one risk factor for metabolic syndrome. And 16 (17%) patients had the diagnosis of metabolic syndrome. Overall, fibrosis stages of the enrolled patients were scored as F0 (n=19), F1 (n=28), F2 (n=16), F3 (n=17), and F4 (n=14). The median degree of steatosis was 10% (range, 5-70%). The patients' characteristics are summarized in Table 1.

### 3.2. Liver Stiffness Measurement (LSM) by TE

**Figure 3:**
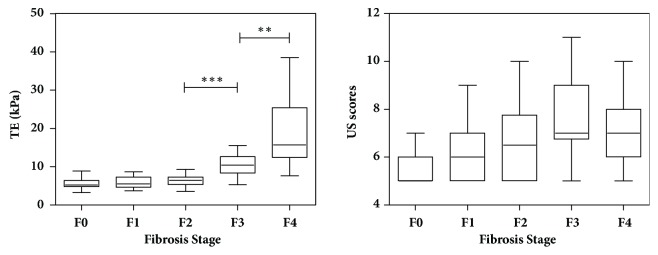
Box and whisker plots of TE and US scores at each fibrosis stage. The central box represents values from lower to upper quartile (25^th^ -75^th^ percentile). The line through each box represents the median. The mean liver stiffness measured with TE increased significantly from F2 to F4 (F2 vs F3, P<0.001; F3 vs F4, P=0.002). ∗∗, P <0.01. ∗∗∗, P <0.001.

TE examination succeeded in 94 patients with CHB and NAFLD. The success rate of TE examination was therefore 100%. As the IQR/LSM ratios were less than 30%, the results of TE measurements were considered to be reliable. Liver stiffness measured with TE ranged from 3.2 to 38.5 kPa (IQR, 5.1-9.5 kPa) (Figure 3, Table 2). The mean LSM was 5.68, 5.83, 6.34, 10.34, and 18.84 for patients with F0, F1, F2, F3, and F4, respectively. The mean LSM gradually increased from F0 to F2 (F0 vs F1, P=0.745; F1 vs F2, P=0.293). Then, the LSM increased significantly from F2 to F4 (F2 vs F3, P<0.001; F3 vs F4, P=0.002).

### 3.3. US Scoring System

Figure 3 shows the distributions of US scores at different fibrosis stages. The interobserver reproducibility of the US scores was 0.93 (95% CI: 0.89, 0.95). The comparison of AUCs revealed that the US scores had superior trends to other measures (the liver surface scores, liver parenchyma scores, hepatic vein contour scores, and the spleen index) in the diagnosis of significant liver fibrosis (AUC, 0.73 vs 0.67, 0.64, 0.67, and 0.51, respectively), in the diagnosis of advanced fibrosis (AUC, 0.76 vs 0.72, 0.63, 0.68, and 0.51, respectively), and in the diagnosis of cirrhosis (AUC, 0.71 vs 0.67, 0.64, 0.58, and 0.51, respectively). Hence, the US scores were selected for further analysis.

### 3.4. Correlations among TE, US, Hepatic Steatosis, and Fibrosis Stage

In the present study, LB was used as the reference standard. The correlation coefficients of TE and US scores with fibrosis stage were 0.69 (95% CI: 0.55, 0.78; P<0.001) and 0.47 (95% CI: 0.30, 0.61; P<0.001), respectively. The correlation coefficients of TE were significantly higher than that of the US scores (P=0.022). However, the degree of hepatic steatosis did not correlate with fibrosis stage (r=0.041, P=0.69), TE scores (r=0.037, P=0.72), or US scores (r=0.091, P=0.38). Next, in order to evaluate the impact of hepatic steatosis on the performance of TE in assessing liver fibrosis, patients were stratified into three groups, group 1 (5%≤hepatic steatosis<10%, n=33), group 2 (10%≤hepatic steatosis<20%, n=26), and group 3 (hepatic steatosis≥20%, n=35). The correlation coefficients of TE with fibrosis stage in different groups were 0.69 (95% CI: 0.46, 0.84; P<0.001), 0.61 (95% CI: 0.30, 0.81; P<0.001), and 0.72 (95% CI: 0.50, 0.85; P<0.001), respectively. However, no significant differences existed among the three groups in correlation coefficient (all P>0.05). Moreover, the comparison of AUCs revealed that no significant differences were found among the three groups in the assessment of liver fibrosis using TE (all P>0.05, Table 3). Taken together, these data indicate that the degree of hepatic steatosis may not influence the performance of TE in the assessment of liver fibrosis.

### 3.5. TE and US Scores for Significant Fibrosis Assessment (F ≥2)

**Figure 4:**
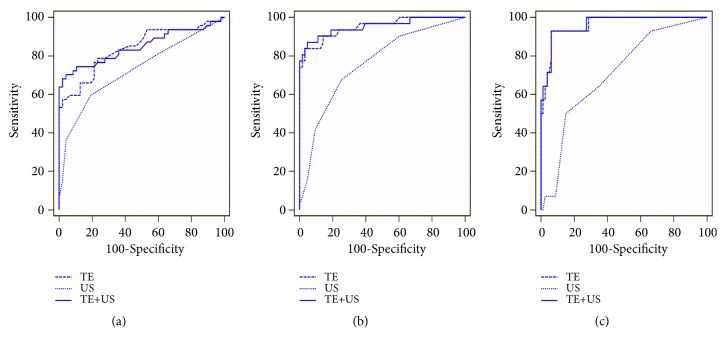
ROC curves of TE, US, and TE combined with US for significant fibrosis assessment (a), advanced fibrosis assessment (b), and cirrhosis assessment (c) in CHB patients concurrent with nonalcoholic fatty liver disease.

Figure 4 shows the diagnostic performance of TE, US, and TE combined with US, as assessed by ROC curves.  Table 4 shows the AUCs and predictive values for significant fibrosis. Comparison of the AUCs revealed that both TE and TE plus US were significantly superior to US in the diagnosis of significant fibrosis (AUC, 0.84 vs 0.73, P=0.02; AUC, 0.85 vs 0.73, P=0.002). Compared with TE alone, combining TE with US did not increase the diagnostic performance of detecting significant fibrosis (AUC, 0.85 vs 0.84, P=0.62). However, their combination significantly increased the specificity (95.7% vs 76.6%, P<0.001) and PPV (94.3% vs 77.1%, P=0.002) compared to TE alone.

### 3.6. TE and US Scores for Advanced Fibrosis Assessment (F ≥3)

The diagnostic performance of TE, US, and TE combined with US in advanced fibrosis, as assessed by ROC curves, is shown in Figure 4.  Table 4 shows the AUCs and predictive values for TE and US. The comparison of AUCs revealed that TE was significantly superior to US in the diagnosis of advanced fibrosis (AUC, 0.95 vs 0.76, P<0.001). Compared with TE alone, combining TE with US did not improve the diagnostic performance of detecting advanced fibrosis (P=0.69). When TE alone was used to predict advanced fibrosis, the PPV and NPV were 92.9% and 92.4%, respectively, when 8.7 kPa was selected as the optimal cut-off value.

### 3.7. TE and US Scores for the Diagnosis of Cirrhosis (F=4)

TE, US, and TE combined with US for the evaluation of subclinical cirrhosis (F=4) were assessed by ROC curves, and the results are shown in Figure 4.  Table 4 shows the AUCs and predictive values for TE and US. The comparison of AUCs revealed that TE was significantly superior to US in diagnosing cirrhosis (AUC, 0.96 vs 0.71, P<0.001). In addition, combining TE with US did not improve the diagnostic performance of detecting liver cirrhosis compared with TE alone (P=0.38). Compared to US, TE had a significantly higher sensitivity (92.9% vs 50.0%; P<0.001) and PPV (72.2% vs 36.8%; P<0.001) in predicting cirrhosis. Moreover, when using the cut-off value of 10.9 kPa, TE showed a high NPV of 98.7% in excluding the diagnosis.

## 4. Discussion

We herein report a single-center experience comparing TE, US, and their combination in the assessment of fibrosis in a cohort of CHB patients with NAFLD. In this study, comparison of AUCs revealed that TE was significantly superior to US in the diagnosis of fibrosis and subclinical cirrhosis. Combining TE with US did not increase the accuracy of detecting significant fibrosis, advanced cirrhosis, or cirrhosis compared to TE alone. However, combining TE with US can significantly increase the PPV in predicting significant fibrosis when compared to TE alone. The optimal cut-off values of TE for advanced fibrosis and cirrhosis were 8.7 kPa and 10.9 kPa, with NPVs of 92.4% and 98.7%, respectively.

HBV infection is a major etiology of chronic liver disease worldwide. In the past decade, NAFLD has emerged as a common liver disorder in the general population [3]. Accordingly, the number of CHB patients with concomitant NAFLD is rapidly growing. Approximately 25% of CHB patients have coexisting NAFLD [21]. In the present study, the rate of NAFLD in CHB patients was estimated to be 24.3%, which is similar to the general incidence, and approximately one-third of the current patients had at least one risk factor for metabolic syndrome. Several reports have revealed that metabolic syndrome can increase the risk of liver fibrosis progression and liver cirrhosis in CHB patients [22, 23]. Since the prognosis of patients and the treatment selection depends on fibrosis stages, accurate assessment of liver fibrosis is urgent.

Although TE may be affected by several factors, it performs well in CHB patients and may reduce the need for LB [12, 24]. The effects of hepatic steatosis on TE performance in patients with chronic HCV and NAFLD may be more definitive, resulting in overestimations of the liver fibrosis stage [25, 26]. However, the role of hepatic steatosis in CHB remains controversial [27, 28]. In the present study, the extent of hepatic steatosis did not correlate with TE, US, or the histological fibrosis stage (all P>0.05). Also, no significant differences existed in correlation coefficient of TE with fibrosis stage among different degrees of hepatic steatosis (all P>0.05). In addition, TE performs at a good to excellent level of accuracy in detecting fibrosis and subclinical cirrhosis, which is similar to the results found in a previous meta-analysis of a large sample of CHB patients [24]. These findings indicate that TE could be useful and reliable in assessing liver fibrosis in CHB patients, even in those patients concurrent with NAFLD. Nevertheless, the exact impact of hepatic steatosis on TE performance requires further evaluation.

A US scoring system consisting of four factors has been developed and has proven to be reliable in evaluating liver fibrosis and cirrhosis [10]. Since US is routinely used to assess structural changes caused by CHB, it is necessary to compare TE with US before introducing TE to evaluate fibrosis in CHB patients with NAFLD. To our knowledge, this study is the first to demonstrate the performance of TE and US in CHB patients with NAFLD. TE proved to be superior to US in the diagnostic performance of predicting significant fibrosis (P=0.02) and advanced fibrosis (P<0.001). Furthermore, a combination of TE and US was equivalent to TE in diagnostic accuracy (P=0.62 and P=0.69). However, TE and US combination increased the PPV from 77.1% to 94.3% in predicting significant fibrosis. These results indicate that TE alone was useful to assess significant fibrosis and advanced fibrosis in CHB patients with NAFLD, but TE combined with US can help predict significant fibrosis.

Cirrhosis is a high-risk factor for developing HCC and complications caused by portal hypertension. Therefore, early detection of subclinical cirrhosis can help identify high-risk patients earlier and start the optimized therapy accordingly. US is a widely available tool for diagnosing cirrhosis, despite its low sensitivity and high specificity. In the present study, the AUC of US in detecting cirrhosis was 0.71, which is relatively lower compared to the results of a previous study (AUC, 0.79) [10]. This variation could be related to differences in the enrolled patients. A previous study mainly enrolled viral patients (HBV and HCV), and only two patients had hepatic steatosis. However, this study recruited only CHB patients with NAFLD. More importantly, TE demonstrated excellent diagnostic performance in predicting subclinical cirrhosis, with high sensitivity and specificity of 92.9% and 93.8%, respectively. Furthermore, the overall diagnostic performance of TE was significantly different from that of US (P<0.001). Compared with TE alone, combining TE with US did not improve the diagnostic performance (P=0.38). These findings are of vital importance because TE is much simpler and safer than LB is. Given the high NPV of TE (98.7%), it is unnecessary to perform LB in patients who are suspected of cirrhosis at US examination. Patients can avoid LB and start an antiviral therapy program earlier. Thus, we suggest that TE should be performed on patients with doubtful cirrhosis at routine US.

The optimum cut-off value selected for predicting a patient's fibrosis stage differs from case to case. Etiology should be taken into account in assessing fibrosis using TE [12]. The optimum cut-off value for F ≥2 was 6.6 kPa in the present study, which was slightly lower than the values for NAFLD (6.9 kPa) and CHB patients (7.2 kPa). However, the optimum cut-off values for F ≥3 and F=4 were 8.7 kPa and 10.9 kPa, which were intermediate values between those for NAFLD (8.4 kPa and 10.3 kPa) and CHB patients (9.4 kPa and 12.2 kPa) [24, 26, 29]. This variation may be explained by the degree of hepatic steatosis. A previous study indicated that hepatic steatosis could influence the architectural structure of the liver, potentially changing the propagation time of the vibratory wave through the liver [30]. Statistical analysis indicated that cut-off values with a PLR >10.0 offer sufficient confidence to confirm the diagnosis, while an NLR <0.1 provides enough confidence to exclude the diagnosis [29]. In our study, the cut-off value of 10.9 kPa for assessing cirrhosis had a PLR of 14.9 and an NLR of 0.08. Thus, using 10.9 kPa as an optimal cut-off value in diagnosing cirrhosis in CHB patients with NAFLD may be suitable. Nevertheless, further studies are warranted to confirm the accuracy.

By definition, NAFLD indicates the absence of significant alcohol consumption. However, the optimal cut-off for significant alcohol consumption in patients with suspected NAFLD remains controversial. It was inconsistent and ranged from >10 g of alcohol per day to >40 g per day [31]. For NASH clinical trials candidate eligibility purposes, significant alcohol consumption was defined as >30 g/day in men and >20 g/day in women [32]. Furthermore, this definition has been recommended by western guidelines, but with weak strength and relatively low quality [33, 34]. While in the Asia-Pacific region, significant alcohol consumption has been defined as >20 g/day for men and >10 g/day for women by the Asia-Pacific Working Party on NAFLD [18] and has been widely used [7, 35]. Moreover, even with consuming moderate amounts of alcohol, patients may be still predisposed to NAFLD if they have metabolic risk factors [34]. Thus, to reduce the confounding impact of alcohol, the lower cut-off of significant alcohol consumption (defined as >20 g/day for men and >10 g/day for women) was used in the present study. It should be noted that this study has several limitations. First, most patients showed mild to moderate hepatic steatosis. The impact of the entire spectrum of steatosis on TE could not be fully assessed. In addition, due to the unavailability of XL probe for TE, patients with BMI ≥30 kg/m^2^ were excluded in the present study. This may represent a selection bias since steatosis is more prevalent and severe in obese patients. Third, as controlled attenuation parameter (CAP) was unavailable in our department until 2018, it was not assessed for comparison with histological or US findings. Moreover, our sample size was relatively small.

In conclusion, although TE has a better performance over US, and over the combination of TE and US, this should not discourage the use of US in CHB patients with NAFLD. TE is more reliable in the assessment of liver fibrosis and can avoid unnecessary liver biopsies. Nevertheless, US is necessary in order to exclude liver focal lesions and to assess the presence of portal hypertension.

## Figures and Tables

**Table 1:** Patients' characteristics.

Characteristic	Standard Value (Range)	Patients (n=94)

Mean age (years)	NA	36.90±8.17
Male gender (n, %)	NA	85 (90.4%)
Body mass index (BMI) (kg/m^2^)	NA	24.20±2.68
BMI ≥ 25 (n, %)	NA	36 (37.9%)
Aspartate aminotransferase (IU/L)	15-40	29 (15-128)
Alanine aminotransferase (IU/L)	3-35	40.5 (9-173)
Total bilirubin (umol/L)	4-23.9	12.7 (5.6-32.5)
Albumin (g/L)	36-51	45.5 (36.2-51.6)
g-Glutamyltransferase (IU/L)	10-60	32.5 (12-283)
Alkaline phosphatase (IU/L)	45-125	72.5 (33-207)
Platelets count (10^3^/mm^3^)	100–350	200.43±54.87
Prothrombin time activity (%)	70–120	96.18±11.68
Fasting glucose (mmol/L)	3.9-6.1	4.90 (3.64-8.61)
Fasting glucose ≥5.6 mmol/L (n, %)	≥5.6	23 (24.5%)
Total cholesterol (mmol/L)	3.1-5.7	4.78±0.84
Triglyceride (mmol/L)	0.34-1.92	1.19 (0.39-4.89)
Triglyceride ≥ 1.7 mmol/L (n, %)	≥1.7	22 (23.4%)
HDL-cholesterol (mmol/L)	0.78-2.00	1.15±0.23
Reduced HDL-cholesterol (mmol/L) (n, %)	<1.03 in men <1.29 in women	29 (30.8%)
LDL-cholesterol (mmol/L)	2.07-3.10	3.17±0.83
Elevated LDL-cholesterol (n, %)	>3.10	52 (55.3%)
HBeAg positive (n, %)	>1	38 (40.4%)
HBV-DNA (log10 IU/mL)	<20 IU/mL	4.83 (1.54-8.68) ^∗^
Ultrasonography score		
4/5	NA	0/28
6/7	NA	29/18
8/≥9	NA	11/8
Fibrosis score (METAVIR)		
F0 (n, %)	NA	19 (20.2%)
F1 (n, %)	NA	28 (29.7%)
F2 (n, %)	NA	16 (17.0%)
F3 (n, %)	NA	17 (18.1%)
F4 (n, %)	NA	14 (15.0%)
Hepatic steatosis		
≥5%	NA	90 (95.7%)
34-66%	NA	3 (3.2%)
>66%	NA	1 (1.1%)

Unless otherwise indicated, data were expressed as means ± standard deviations or medians and ranges. ^∗^Eleven patients had undetectable HBV-DNA loads. HDL=high density lipoprotein; LDL= low-density lipoprotein; NA=not applicable.

**Table 2:** Distribution of liver stiffness measured by TE at different fibrosis stages.

TE (kPa) and P value	F0 (n=19)	F1 (n=28)	F2 (n=16)	F3 (n=17)	F4 (n=14)
Mean value^∗^	5.68±1.48	5.83±1.52	6.34±1.61	10.34±2.91	18.84±8.35
Median value^†^	5.2 (3.2-8.9)	5.5 (3.7-8.7)	6.5 (3.5-9.3)	10.4(5.3-15.5)	15.7 (7.6-38.5)
P value^‡^	Not applicable	0.745	0.293	<0.001	0.002

^*∗*^Data were expressed as means ± standard deviations.

^†^Data were expressed as medians and ranges.

^‡^Mean value compared with the next lower fibrosis stage.

**Table 3:** Diagnostic performances of TE in patients with different degrees of hepatic steatosis for liver fibrosis and cirrhosis assessment.

Statistic	5% ≤HS<10% (n=33)	10%≤HS<20% (n=26)	HS ≥20% (n=35)

**Significant fibrosis assessment (F≥2)**			
AUC	0.78 (0.60, 0.90)	0.79 (0.59, 0.93)	0.93 (0.79, 0.99)
Standard error	0.083	0.092	0.041
Cut-off value	6.6	6.8	8.4
Sensitivity (%)	83.3 (58.6, 96.4)	66.7 (34.9, 90.1)	76.5 (50.1, 93.2)
Specificity (%)	66.7 (38.4, 88.2)	85.7 (57.2, 98.2)	100.0 (81.5, 100.0)
PPV (%)	75.0 (50.9, 91.3)	80.0 (44.4, 97.5)	100.0 (75.3, 100.0)
NPV (%)	76.9 (46.2, 95.0)	75.0 (47.6, 92.7)	81.8 (59.7, 94.8)
Comparison of AUCs			
5% ≤HS<10%	-	P=0.94	P=0.11
10%≤HS<20%	P=0.94	-	P=0.16
HS ≥20%	P=0.11	P=0.16	-
**Advanced fibrosis assessment (F≥3)**			
AUC	0.96 (0.83, 0.99)	0.94 (0.77, 0.99)	0.95 (0.82, 0.99)
Standard error	0.037	0.054	0.039
Cut-off value	8.6	7.2	8.4
Sensitivity (%)	90.0 (55.5, 99.7)	85.7 (42.1, 99.6)	85.7 (57.2, 98.2)
Specificity (%)	95.7 (78.1, 99.9)	89.5 (66.9, 98.7)	95.2 (76.2, 99.9)
PPV (%)	90.0 (55.5, 99.7)	75.0 (34.9, 96.8)	92.3 (64.0, 99.8)
NPV (%)	95.7 (78.1, 99.9)	94.4 (72.7, 99.9)	90.9 (70.8, 98.9)
Comparison of AUCs			
5% ≤HS<10%	-	P=0.76	P=0.85
10%≤HS<20%	P=0.76	-	P=0.88
HS ≥20%	P=0.85	P=0.88	-
**Cirrhosis assessment (F=4)**			
AUC	1.00 (0.89, 1.00)	0.98 (0.79, 1.00)	0.90 (0.75, 0.98)
Standard error	0.000	0.029	0.076
Cut-off value	10.7	10.0	10.9
Sensitivity (%)	100.0 (59.0, 100.0)	100.0 (15.8, 100.0)	80.0 (28.4, 99.5)
Specificity (%)	100.0 (86.8, 100.0)	95.8 (78.9, 99.9)	86.7 (69.3, 96.2)
PPV (%)	100.0 (59.0, 100.0)	66.7 (9.4, 99.2)	50.0 (15.7, 84.3)
NPV (%)	100.0 (86.8, 100.0)	100.0 (85.2, 100.0)	96.3 (81.0, 99.9)
Comparison of AUCs			
5% ≤HS<10%	-	P=0.49	P=0.19
10%≤HS<20%	P=0.49	-	P=0.33
HS ≥20%	P=0.19	P=0.33	-

Data in parentheses were 95% confidence interval. AUC=area under the ROC curve. HS=hepatic steatosis. PPV=positive predictive value. NPV=negative predictive value.

**Table 4:** Diagnostic performances of TE, US, and TE combined with US in CHB patients with NAFLD for liver fibrosis and cirrhosis assessment.

Statistic	TE	US	TE plus US

**Significant fibrosis assessment (F≥2)**			
AUC	0.84 (0.75, 0.91)	0.73 (0.63, 0.82)	0.85 (0.76, 0.92)
Standard error	0.042	0.051	0.043
Cut-off value	6.6	6	0.61
Sensitivity (%)	78.7 (64.3, 89.3)	59.6 (44.3, 73.6)	70.2 (55.1, 82.7)
Specificity (%)	76.6 (62.0, 87.7)	80.9 (66.7, 90.9)	95.7 (85.5, 99.5)
PPV (%)	77.1 (62.7, 88.0)	75.7 (58.8, 88.2)	94.3 (80.8, 99.3)
NPV (%)	78.3 (63.6, 89.1)	66.7 (52.9, 78.6)	76.3 (63.4, 86.4)
Positive LR	3.36 (2.0, 5.8)	3.11 (1.7, 5.9)	16.50 (4.2, 64.9)
Negative LR	0.28 (0.2, 0.5)	0.50 (0.3, 0.7)	0.31 (0.2, 0.5)
**Advanced fibrosis assessment (F≥3)**			
AUC	0.95 (0.89, 0.98)	0.76 (0.66, 0.84)	0.95 (0.89, 0.99)
Standard error	0.024	0.052	0.026
Cut-off value	8.7	6	0.39
Sensitivity (%)	83.9 (66.3, 94.5)	67.7 (48.6, 83.3)	87.1 (70.2, 96.4)
Specificity (%)	96.8 (89.0, 99.6)	74.6 (62.1, 84.7)	95.2 (86.7, 99.0)
PPV (%)	92.9 (76.5, 99.1)	56.8 (39.5, 72.9)	90.0 (73.5, 97.9)
NPV (%)	92.4 (83.2, 97.5)	82.5 (70.1, 91.3)	93.7 (84.8, 98.3)
Positive LR	26.4 (6.7, 104.2)	2.7 (1.6, 4.3)	18.3 (6.0, 55.6)
Negative LR	0.17 (0.07, 0.4)	0.43 (0.3, 0.7)	0.14 (0.05, 0.3)
**Cirrhosis assessment (F=4)**			
AUC	0.96 (0.90, 0.99)	0.71 (0.61, 0.80)	0.96 (0.90, 0.99)
Standard error	0.023	0.071	0.022
Cut-off value	10.9	7	0.14
Sensitivity (%)	92.9 (66.1, 99.8)	50.0 (23.0, 77.0)	92.9 (66.1, 99.8)
Specificity (%)	93.8 (86.0, 97.9)	85.0 (75.3, 92.0)	93.8 (86.0, 97.9)
PPV (%)	72.2 (46.5, 90.3)	36.8 (16.3, 61.6)	72.2 (46.5, 90.3)
NPV (%)	98.7 (92.9, 100.0)	90.7 (81.7, 96.2)	98.7 (92.9, 100.0)
Positive LR	14.9 (6.3, 35.1)	3.3 (1.6, 7.0)	14.9 (6.3, 35.1 )
Negative LR	0.08 (0.01, 0.5)	0.59 (0.3, 1.0)	0.08 (0.01, 0.5)

Data in parentheses were 95% confidence interval. AUC=area under the ROC curve. PPV=positive predictive value. NPV=negative predictive value. LR=likelihood ratio.

## Data Availability

The data used or analyzed during the current study are available from the first author (Geng-lin Zhang) on reasonable request.

## Abbreviations

CHB: Chronic hepatitis B
NAFLD: Nonalcoholic fatty liver disease
LB: Liver biopsy
TE: Transient elastography
US: Ultrasonography
ROC curve: Receiver operating characteristic curve
AUCs: The areas under the ROC curves.
